# Kaempferitrin Attenuates Lipopolysaccharide‐Induced Cardiac Dysfunction Through Suppression of the NF‐κB/NLRP3 Signaling Pathway

**DOI:** 10.1002/iid3.70323

**Published:** 2026-01-26

**Authors:** Hongyu Kuang, Qiang Li, Min Chen, Huaan Du

**Affiliations:** ^1^ Department of Cardiology University‐Town Hospital of Chongqing Medical University Chongqing China; ^2^ Department of Cardiology Children's Hospital of Chongqing Medical University Chongqing China; ^3^ Department of Pediatrics Women and Children's Hospital of Chongqing Medical University Chongqing China

**Keywords:** cardiac dysfucntion, Kaempferitrin, lipopolysaccharide, NF‐κB signaling pathway

## Abstract

**Purpose:**

The inflammatory activation and metabolic disorders of cardiomyocytes are essential mechanisms in sepsis‐related cardiac dysfunction. Kaempferitrin (Kae), a flavonoid compound, possesses various properties including anti‐inflammatory and anti‐glycation effects. Hence, the current study is conducted to investigate the protective effects of Kae against sepsis‐induced cardiac dysfunction.

**Methods:**

C57BL/6 J mice were treated with Kae for 2 h, followed by lipopolysaccharide (LPS) treatment. After 12 h, the echocardiographic measurements were conducted. Serum test, pathological analysis, transcriptomics, western blotting, and RT‐PCR were used for exploring mechanisms. Additionally, in vitro, H9c2 and AC16 cardiomyocyte cell lines were pretreated with Kae (10 μM) for 2 h, followed by LPS stimulation (1 μg/mL).

**Results:**

In vivo, pretreatment with Kae mitigates LPS‐induced cardiac dysfunction. Kae suppresses the levels of IL‐6, TNF‐α, IL‐1β, and IL‐18 in the cardiac tissue of mice mediated by LPS. Additionally, serological and histological assessments demonstrate that Kae exhibits protective effects against LPS‐induced cardiomyocyte injury and apoptosis. Transcriptomic analysis reveals that the nuclear factor kappa‐B (NF‐κB)/NLRP3 signaling pathway may be a crucial mechanism. Meanwhile, it proved that LPS significantly activates NF‐κB/NLRP3 to induce cardiomyocyte pyroptosis, which is attenuated by Kae. In *vitro*, H9c2 and AC16 cardiomyocyte cell lines were pretreated with Kae followed by LPS stimulation, showing an inhibition of NF‐κB/NLRP3 pathway, with a decreased mRNA levels of *Il‐6*, *Tnf‐α*, *Il‐1β*. The NLRP3‐knock out (*Nlrp3* ^*−*/*−*^) mice have verified that Kae ameliorating LPS‐induced spetic cardiomyopathy by inhibiting NLRP3.

**Conclusions:**

This study confirms that Kae alleviates LPS‐induced left ventricular remodeling and cardiac dysfunction by suppressing the NF‐κB/NLRP3/pyroptosis pathway.

**Abbreviations**
CCK‐8: cell counting kit‐8
CK‐MB: creatine kinase‐MB
DMEM: Dulbecco's modified Eagle's medium
FBS: fetal bovine serum
GSDMD: gasdermin D
IL‐6: interleuckin‐6
Kae: Kaempferitrin
LDH: lactate dehydrogenase
LPS: lipopolysaccharide
LVEF: left ventricular ejection fraction
LVFS: left ventricular fractional shortening
LVIDd: left ventricular internal diameter at diastole
LVIDs: left ventricular internal diameter at systole
NO: nitric oxide
PCR: polymerase chain reaction
TNF‐α: tumor necrosis factor‐α
TUNEL: TdT‐mediated dUTP nick end‐labeling

## 1 Introduction

Sepsis is a syndrome caused by a series of overly activated immune responses, eventually leading to multiple organ dysfunctions (MODFs) without prompt treatment [1]. A substantial body of research has identified a high prevalence of cardiac dysfunction following sepsis, with reported incidence rates ranging from 20% to 60% [2]. As demonstrated in previous studies [3, 4], these patients frequently exhibit elevated mortality rates. Consequently, the amelioration of cardiac dysfunction induced by sepsis significantly contributes to the reduction of mortality.

At the cellular and molecular levels, previous studies have indicated that the inflammatory activation [5, 6], in addition to the metabolic disorders of cardiomyocytes represents a significant mechanism in sepsis‐related cardiac dysfunction. It can thus be concluded that reducing the levels of inflammatory factors in cardiomyocytes has the potential to improve cardiac function. Lipopolysaccharide (LPS), a bacterial endotoxin, has been demonstrated to instigate excessive inflammatory responses, resulting in acute damage and dysfunction of multiple organs. This phenomenon has been widely recognized as a model of sepsis on a global scale [7].

Kaempferitrin (Kae), a predominant natural flavonoid glycoside found in Bauhinia forficata leaves, has been demonstrated to exhibit a range of pharmacological properties in diabetes and tumors [8, 9, 10, 11]. At the present time, there is an absence of scientific evidence to support the hypothesis that Kae has an effect on cardiovascular disorders. It is hypothesized that Kae may be effective in cases of myocardial damage induced by inflammation infiltration, particularly in cases of sepsis‐induced cardiac dysfunction, due to its significant anti‐inflammatory properties.

The present study has been conducted for the purpose of investigating the effects of Kae on sepsis‐induced cardiac dysfunction, as well as for the exploration of its underlying mechanisms of action.

## 2 Methods and Materials

### 2.1 Animals and Groups

All animal experimental procedures were conducted in accordance with the Guidelines for the Care and Use of Laboratory Animals and were approved by the Ethics Committee of the Animal Experimental Center at Chongqing Medical University. The experiment was conducted using male C57BL/6 J mice, which were 6–8 weeks of age and weighed between 20 and 25 g. The mice were procured from Huachuang Sino Pharmaceutical Technology Co. Ltd. (Jiangsu, China). The minimal number of mice required per group to complete the study was six. All animals were housed in a specific pathogen‐free and environmentally controlled facility. Following an adaptive feeding period spanning 1 week, the mice were then randomly divided into five groups, including: The experiment comprised six rats in each of the following groups: (1) Control; (2) Kae (20 mg/kg, MCE #no. HY‐N0628, purity: 99.94%); (3) LPS (Sigma‐Aldrich, #L2630, 10 mg/kg) [12]; (4) LPS + Kae (10 mg/kg); and (5) LPS + Kae (20 mg/kg) [8, 13]. The investigation was conducted to ascertain the effect of Kae. Mice were treated with Kae 2 h prior to undergoing a 12‐h intraperitoneal injection of LPS [5, 6, 7].

The NLRP3 knock‐out (*Nlrp3* ^KO^) mice were procured from GemPharamtech Co. Ltd (Jiangsu, China). The study comprised four groups: (1) WT + Vehicle (*n* = 6); (2) WT + Kae (*n* = 6); (3) *Nlrp3* ^KO^ + Vehicle (*n* = 6); (4) *Nlrp3* ^KO^ + Kae (*n* = 6). The present study investigates the effects of Kae on a murine model of endotoxemia. A total of 24 mice were treated with LPS stimulation, and to investigate the effect of Kae, mice were also treated with Kae 2 h prior to a 12‐h intraperitoneal injection of LPS.

All mice in the current study were sacrificed after the measurements by echocardiography. The random number table was used to conduct the randomization, and the procedures and analyses were performed in a blinded manner.

### 2.2 Echocardiography

Prior to undergoing echocardiographic examination, the mice were anaesthetized with 2.5% isoflurane. Two‐dimensional‐guided M‐mode echocardiographic images at the parasternal long‐axis were recorded. A series of echocardiographic parameters were measured by three to five consecutive heartbeats, with the procedure conducted blindly. The parameters included left ventricular internal diameter at diastole (LVIDd), left ventricular internal diameter at systole (LVIDs), and left ventricular wall thickness. Subsequently, the left ventricular ejection fraction (LVEF) and left ventricular fractional shortening (LVFS) were calculated based on these data.

### 2.3 Biochemical Analysis

Following the collection of blood samples, centrifugation was performed at 2000 rpm for 20 min at 4°C. The resulting serum was then frozen and stored at −80°C for subsequent analysis. Serum levels of creatine kinase‐MB (CK‐MB) and lactate dehydrogenase (LDH) were measured by means of commercial kits (Servicebo, China).

### 2.4 ELISA Assay

The contents of the interleuckin‐1β (IL‐1β, EM0109, FineTest, China) and interleukin‐6 (IL‐6, EM0121, FineTest, China), tumor necrosis factor‐α (TNF‐α, EM0183, FineTest, China), and IL‐18 (EM1158, FineTest, China) in heart tissues from mice were detected by an ELISA kit according to the manufacturer's instructions.

### 2.5 Histological Morphological Features

The hearts were meticulously excised and then fixed overnight in 4% paraformaldehyde at room temperature. This was followed by dehydration through an alcohol gradient and embedding in paraffin. Cardiac tissues were embedded in paraffin and sectioned at 3–5 μm intervals. The cross‐sections were subjected to haematoxylin and eosin (H&E) staining, and subsequently visualized by light microscopy.

### 2.6 TdT‐Mediated dUTP Nick End‐Labeling (TUNEL) Staining

The apoptosis of heart tissue slices from different groups were detected by the TUNEL kit (Servicebio, China) according to the protocol, and the images merged with a visualization of nuclei using 2‐(4‐amidinophenyl)‐6‐indolecarbamidine dihydrochloride were captured via a fluorescence microscope. The images were captured using a fluorescence microscope and analyzed using Image‐Pro Plus (IPP, version 6.0). The level of apoptosis was determined as the percentage of TUNEL‐positive nuclei to that of the total nuclei.

### 2.7 RNA‐Sequencing and Data Analysis

Samples from three tests selected from two groups, including LPS + Kae (20 mg/kg) and LPS, were sent for mRNA sequencing analysis. The sequencing was performed on an Illumina HiSeq. 2500 platform at LC‐Bio Technologies Co. Ltd. (Hangzhou, China). Cutadapt was employed to eliminate adapter contaminants, low‐quality bases, and undetermined bases from the reads. Subsequently, the quality of the sequence was verified using the FastQC tool. The differentially expressed mRNAs with a log2 (fold change) of at least 1 or log2 (fold change) of at least −1 and a *p*‐value of less than 0.05 were selected for further analysis.

### 2.8 Cell Cultures and Treatment

The H9c2 cell line and AC16 cell line under investigation were procured from Procell (Wuhan, China). The cells were cultivated in Dulbecco's modified Eagle's medium (DMEM, Gibco) with 4.5 g/L glucose, 10% fetal bovine serum (FBS, Gibco), and 1% penicillin/streptomycin. The cells were then placed in a humidified incubator with 5% CO2 at 37°C. H9c2 cells were exposed to varying concentrations of Kae (5, 10, 25, 50 μM) for a duration of 12 h, and the resultant cell viability was detected utilizing the Cell Counting Kit‐8 (CCK‐8) assay (Biyotime, China) in accordance with the manufacturer's instructions. The percentage of Kae‐treated cells was calculated to determine the optimal concentration. Subsequent determination revealed that 10 μM represented the optimal concentration of Kae. The cells were then incubated with DMEM, Kae (10 μM), LPS (1 μg/mL) [14], and LPS (1 μg/mL) + Kae (10 μM) (pretreated Kae for 2 h followed by LPS incubation). To evaluate the impact of pyroptosis in Kae‐mediated cardioprotection against LPS‐induced cardiotoxicity, the caspase‐1 inhibitor VX765 (MCE, #HY‐13205, 25 μM) [15] was utilized in accordance with the manufacturer's instructions.

### 2.9 Western Blotting

Proteins from heart tissues and cardiomyocytes were extracted with RIPA buffer (P0013C, Beyotime Biotechnology, Shanghai, China). Following extraction, the samples were loaded onto a 10% separation gel for SDS‐PAGE. Subsequently, the separated proteins were transferred onto a polyvinylidene fluoride membrane. Following the blocking of the membrane with 5% skimmed milk for a period of 1 h at room temperature, the membrane was then subjected to an overnight incubation with a primary antibody at 4°C, including NLRP3 (Adipogen, #AG‐20B‐0014‐C100, 1:1000), p‐p65 (Proteintech, #82335‐1‐RR, 1:1000), p65 (Proteintech, #10745‐1‐AP,1:1000), IkBα (Zenbio, #380682, 1:1000), GSDMD (Abcam, #ab219800, 1:1000), p‐IkBα (Zenbio, #340776, 1:1000), Cleaved‐Caspase 1 (Adipogen,#AG‐20B‐0042‐C100, 1:1000), and GAPDH (Servicebio, #GB15004, 1:1000, 1:1000). The analysis of protein expression levels were normalized by GAPDH using Image J. The protein levels were normalized to 1 value from the control group.

### 2.10 Real‐Time Quantitative Polymerase Chain Reaction (PCR)

Total RNA was isolated from heart tissue using a purified SteadyPure Universal RNA Extraction Kit (AG21022, Accurate Biology, China) and reverse‐transcribed to cDNA using an Evo M‐MLV RT‐PCR Kit (AG11728, Accurate Biology, China). Quantitative PCR was carried out using SYBR Green reagent kits (AG11701, Accurate Biology, China). The Primer sequences used for qPCR are listed in Supplementary Table S1, and the mRNA levels were normalized to *Gapdh*. The mRNA levels were normalized to 1 value from the control group.

### 2.11 Statistical Analysis

The data are presented as mean ± standard error (SEM). The exact group size (number) for each experiment is provided, and number refers to biological replicates, not technical replicates. Differences between two groups were evaluated by unpaired Student's *t*‐test, and one‐way analysis of variance (ANOVA) followed by Bonferroni post hoc test for comparing multiple groups. A statistically significant difference was obtained at *p* < 0.05. All data analyses were implemented in GraphPad Prism 9.0 software (San Diego, CA, USA).

## 3 Results

### 3.1 In vivo, Kae Effectively Attenuated LPS‐Induced Cardiac Injury and Dysfunction

**Figure 1:**
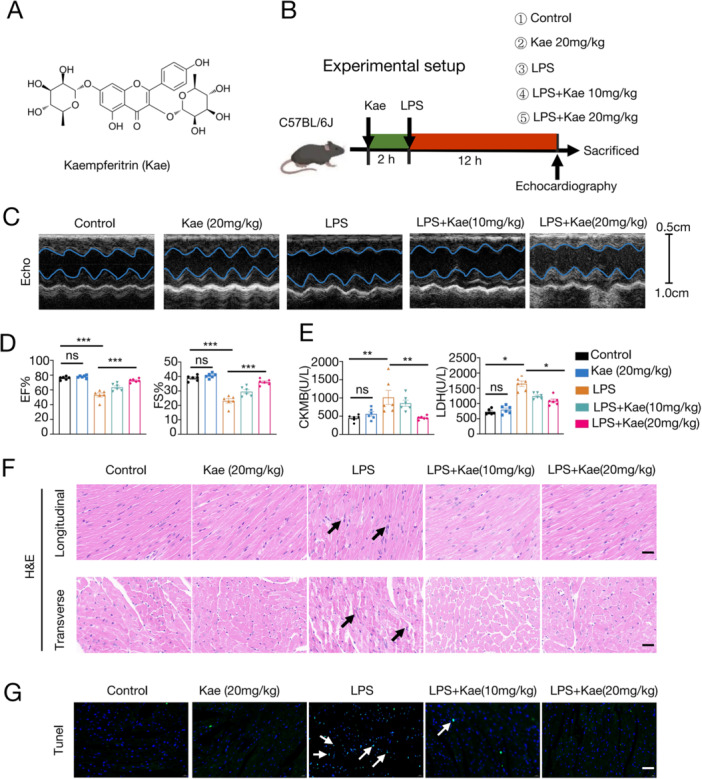
Kaempferitrin inhibits LPS‐induced cardiac inflammation and improves myocardial injury and cardiac function in mice. (A) The structure of Kaempferitrin. (B) The brief experimental setup of the in vivo experiment. After intraperitoneal injection of Kaempferitrin for 2 h, mice were then exposed to LPS (10 mg/kg) for 12 h. (C) Representative transthoracic echocardiography M‐model images from each group. (D) EF% and FS% of the mice in each group. (E) Serum levels of CK‐MB and LDH in mice from each group. (F) Representative images of HE staining for heart tissue. Scale bar = 50 μm. (G) Representative images of TUNEL staining for heart tissue. Scale bar = 50 μm. *N* = 6. (One‐way analysis of variance [ANOVA] followed by Bonferroni post hoc test for comparing multiple groups. *p < 0.05, **p < 0.01, ***p < 0.001, ns = not significant).

The pathological mechanisms underlying LPS‐induced cardiac dysfunction primarily consist of the induction of cardiac inflammation and the subsequent apoptosis of myocardial cells. In order to assess the impact of Kae on LPS‐induced cardiac function, mice were pretreated with Kae at different concentrations (10 and 20 mg/kg), followed by the intraperitoneal injection of LPS (10 mg/kg) (see Figure 1A,B). Echocardiography was conducted to evaluate the cardiac structure and function after 12 h (see Figure 1C, Table S1).

As demonstrated in Figure 1D, the administration of LPS resulted in a significant impairment in cardiac function, as evidenced by the reduced LVEF and LVFS. Moreover, the impairment of cardiac function was mitigated by low‐concentration Kae administration (10 mg/kg), with a greater effect observed at higher concentrations (20 mg/kg). Furthermore, cardiac injury was evidenced by elevated serum levels of LDH and CK‐MB. The outcomes demonstrated that Kae markedly antagonized LPS‐induced myocardial injury, with a dose‐dependent effect (Figure 1E). As demonstrated in Figure 1F, the administration of LPS resulted in significant myocardial dysfunction, tissue rupture, and edema, accompanied by substantial infiltration of inflammatory cells. The ELISA analysis of inflammatory markers in cardiac tissue demonstrated that LPS significantly increased the levels of IL‐6 (see Supporting Information: Figure S1A) and TNF‐α (see Supporting Information: Figure S1B) in the cardiac tissues of mice. However, these increases were significantly alleviated after administration of Kae. Tunnel staining further corroborated the finding that Kae incubation significantly mitigated LPS‐induced apoptosis of cardiomyocytes (see Figure 1G, Supporting Information: Figure S2). Consequently, these data provide evidence that Kae could attenuate LPS‐induced cardiac damages and dysfunction in mice.

### 3.2 Kae Suppressed LPS‐Induced Inflammation and Inhibiting NF‐κB/NLRP3/pyroptosis Pathway In Vitro

**Figure 2:**
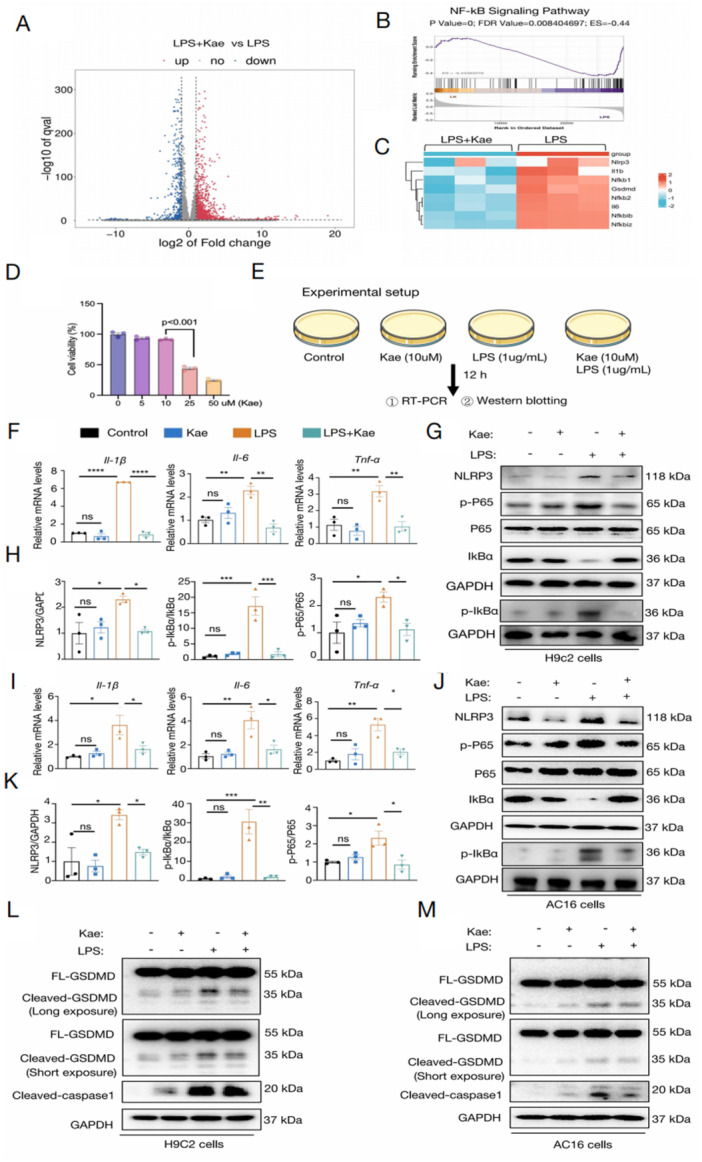
Kae inhibited LPS‐induced cardiomyocyte inflammation and the activation of NF‐κB/NLRP3/pyroptosis pathway in vitro. (A–C) Transcriptome sequencing of myocardial tissue from LPS group and LPS + Kae group. It showed a volcano plot (A), GSEA analysis of NF‐κB pathway (B), and a heat map of genes involved in pyroptosis and NF‐κB signaling pathway (C), *N* = 3. (D) CCK8 assay evaluating the appropriate concentration of Kaempferitrin Kae). (E) The brief experimental setup of the in *vitro* experiment. After maltreatment with Kaempferitrin for 2 h, cardiomyocytes were then exposed to LPS (1 μg/L) for 12 h. (F) The relative RNA levels of *Il‐1β, Il‐6*, and *Tnf‐*α in H9c2 cells. (G) The relative protein levels of NLRP3, p65, p‐p65, Ik‐Bα, p‐Ik‐Bα and GAPDH in H9c2 cell were detected using western blot assay. (H) The densitometric quantification of NLRP3, p‐Ik‐Bα/Ik‐Bα and p‐P65/P65 in H9c2 cells. (I) The relative mRNA levels of *Il‐1β*, *Il‐6*, and *Tnf‐α* in AC16 cells. (J) The relative protein levels of NLRP3, p65, p‐p65, Ik‐Bα, p‐Ik‐Bα, and GAPDH in AC16 cell were detected using western blot assay. (K) Densitometric quantification of NLRP3, p‐Ik‐Bα/Ik‐Bα, and p‐P65/P65 in AC16 cells. (L, M) The relative protein levels of Cleaved‐GSDMD, Cleaved‐caspase1, and GAPDH in H9c2 cells (L) and AC16 cells (M) were detected using western blot assay. *N* = 3. (One‐way analysis of variance [ANOVA] followed by Bonferroni post hoc test for comparing multiple groups. *p < 0.05, **p < 0.01, ***p < 0.001, **** *p* < 0.0001, ns = not significant).

Inflammation is thought to be the main mechanism involved in cardiac injury and dysfunction. To explore the mechanism underlying Kae administration for LPS‐induced cardiac dysfunction, the RNA‐sequencing analysis has been utilized. As indicated by further bioinformatics analysis with gene set enrichment analysis (GSEA) and heatmap visualization, nuclear factor kappa‐B (NF‐κB) signaling pathway was significantly inhibited after Kae supplementation (Figure 2A–C). In vitro, we identified Kae at a concentration of 10 μM as the optimal dose (Figure 2D, Supporting Information: Figure S3). Further, RT‐PCR analysis and immunoblotting were utilized to detect inflammation levels, and NF‐kB/NLRP3/pyroptosis pathway (Figure 2E). After LPS stimulation (1 μg/mL, 12 h), mRNA levels of *Il‐1β*, *Il‐6*, and *Tnf‐α* were showed markedly over expressed in both H9c2 cells (Figure 2F). In accordance with mRNA levels, western blotting was also employed to investigate the protein levels of NF‐κB/NLRP3 pathway. It revealed that LPS significantly elevated the expression levels of p‐p65 and NLRP3 in cardiomyocytes, whereas suppressed expression of IkBα associated with an enhanced phosphorylation level of IkBα. And Kae treatment greatly decrease the protein levels of p‐p65, NLRP3, and p‐IkBα (Figure 2G,H). Moreover, we utilized AC16 cells observing similar results (Figure 2I,J).

Herein, in order to explore the role of pyroptosis in Kae administration in LPS‐induced cardiomyocyte injury, we further confirmed that the expressions of cleaved‐GSDMD, and cleaved‐caspase 1 in cultured cardiomyocytes were prominently increased, which were abolished by Kae incubation in both H9c2 cells and AC16 cells (Figure 2L,M).

### 3.3 Kae Inhibited NF‐κB/NLRP3 Pathway Overactived By LPS in Mice

**Figure 3:**
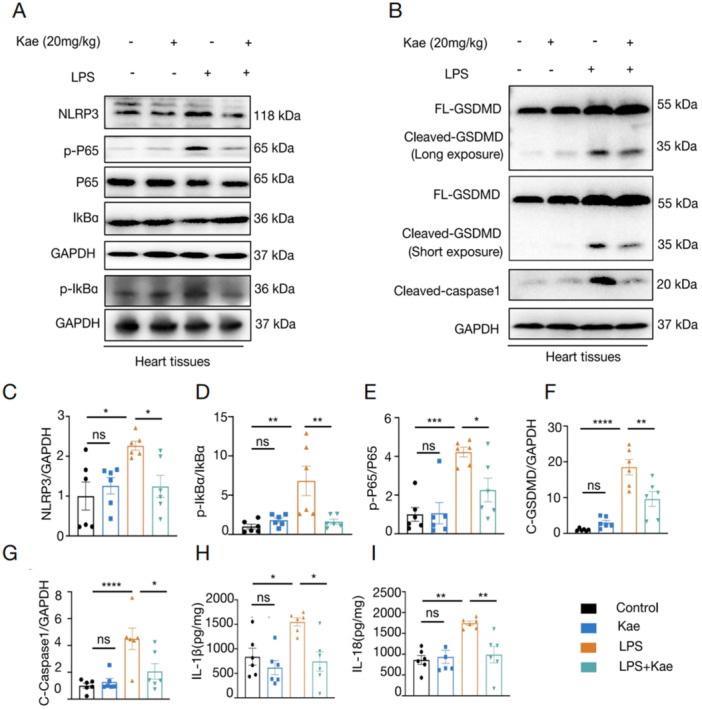
Kaempferitrin may ameliorate LPS‐induced cardiac injury by inhibiting pyroptosis *via* the NF‐κB/NLRP3 pathway in vivo*.* (A) The relative protein levels of NLRP3, P65, p‐P65, IkBα, p‐IkBα, and GAPDH in the heart tissue were detected using western blot assay. (B)The relative protein levels of GSDMD, Cleaved‐caspase1, and GAPDH in the heart tissue were detected using western blot assay. C‐E, Densitometric quantification of NLRP3 (C), p‐IkBα/IkBα (D), and PP65/P65 (E). (F, G) Densitometric quantification of Cleaved‐GSDMD and Cleaved‐caspase 1. (H, I) Levels of IL‐1β (H), IL‐18 (I) in heart tissue detected by ELISA assay. *N* = 6. (One‐way analysis of variance [ANOVA] followed by Bonferroni post hoc test for comparing multiple groups. *p < 0.05, **p < 0.01, ***p < 0.001, ****p < 0.0001, ns = not significant).

At the same time, *Nlrp3* expressions were indicated as low expression in hearts from mice after Kae treating LPS‐induced cardiac injury. Subsequently, as shown by immunoblotting results, LPS injection caused significantly elevated protein levels of NLRP3, phosporylated‐P65, and p‐IkBα levels, which were reversed after Kae treatment (Figure 3A,C–E).

Given that NLRP3 is a critical factor in pyroptosis, immunoblotting results proved that LPS resulted in a prominently increased expression of cleaved‐gasdermin D (GSDMD), cleaved‐caspase 1, which was blunted after an administration with Kae (Figure 3B,F,G). The activation of capase 1‐dependent pyroptosis commonly is associated with release of inflammatory cytokines, including IL‐1β and IL‐18. ELISA assay of cardiac tissues showed that LPS significantly resulted in an enhanced elevation of both IL‐1β (Figure 3H) and IL‐18 (Figure 3I), whereas Kae administration reduced infiltrated levels of these inflammatory cytokines. Collectively, these data suggest that Kae administration inhibited the overactivation of NF‐κB/NLRP3/pyroptosis pathway.

**Figure 4:**
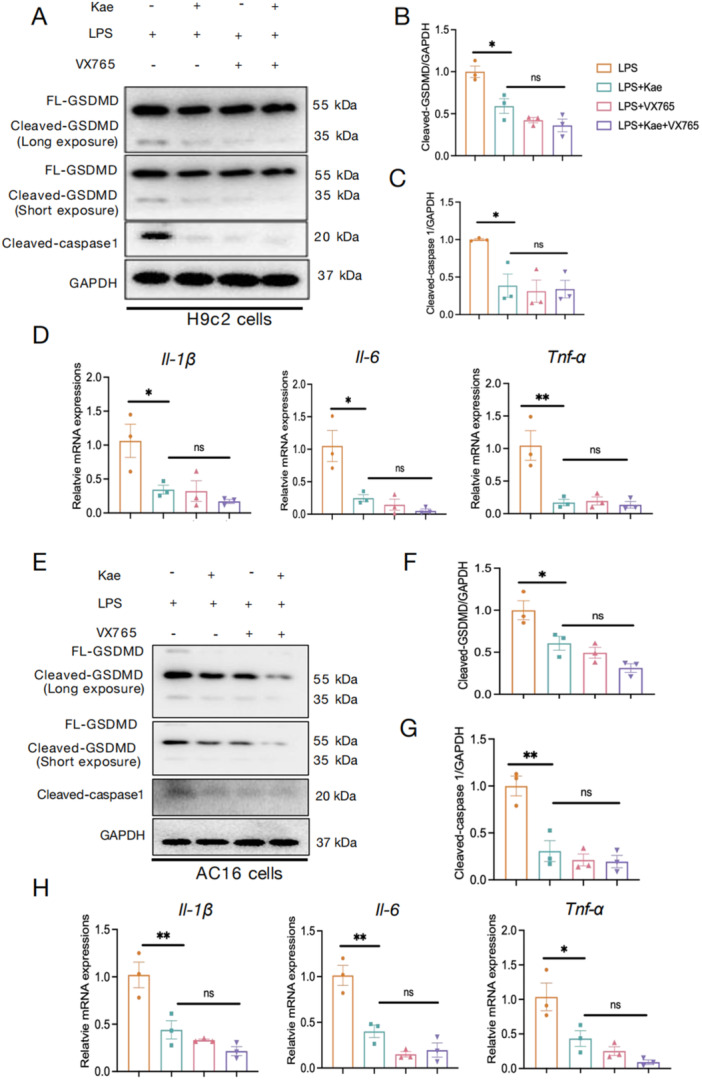
Kaempferitrin improves LPS‐induced myocardial inflammatory damage by inhibiting the NF‐κB/NLRP3/pyroptosis pathway. After pretreatment with Kaempferitrin, VX765 or Kaempferitrin+VX765 for 2 h, cardiomyocytes were then exposed to LPS (1 μg/mL) for 12 h. (A) The relative protein levels of GSDMD, Cleaved‐caspase1 and GAPDH in H9c2 cell were detected using western blot assay. The densitometric quantification of Cleaved‐GSDMD (B), and Cleaved‐Caspase1 (C) in H9c2 cells. (D) The relative mRNA levels of *Il‐1β*, *Il‐6*, and *Tnf‐α* in H9c2 cells. (E) The relative protein levels of GSDMD, Cleaved‐caspase1, and GAPDH in AC16 cell were detected using western blot assay. The densitometric quantification of Cleaved‐GSDMD (F) and Cleaved‐Caspase1 (G) in AC16 cells (H). *N* = 3. (One‐way analysis of variance [ANOVA] followed by Bonferroni post hoc test for comparing multiple groups. *p < 0.05, **p < 0.01, ns = not significant).

When further using the pyroptosis inhibitor VX765, it observed that VX765 treatment suppressed the protein levels of cleaved‐GSDMD and cleaved‐Caspase 1 with LPS incubation, regardless of the treatment of Kae (Figure 4A,B). Moreover, RT‐PCR analysis was carried out to assess the expression levels of inflammatory factors, revealing that VX765 administration also resulted in a significant reduction in mRNA levels of *Il‐1β, Il‐6*, and *Tnf‐α*. Notably, no statistically significant differences were observed among the LPS + Kae group, the LPS + Kae + VX765 group, and the VX765‐treated group (Figure 4C–H). Therefore, it can be inferred that Kae primarily attenuated inflammatory damage in cardiac myocytes by mitigating the level of pyroptosis induced by LPS.

### 3.4 NLRP3 Is Required for Kae to Defend Against Cardiotoxicity Induced by LPS In Vivo

**Figure 5:**
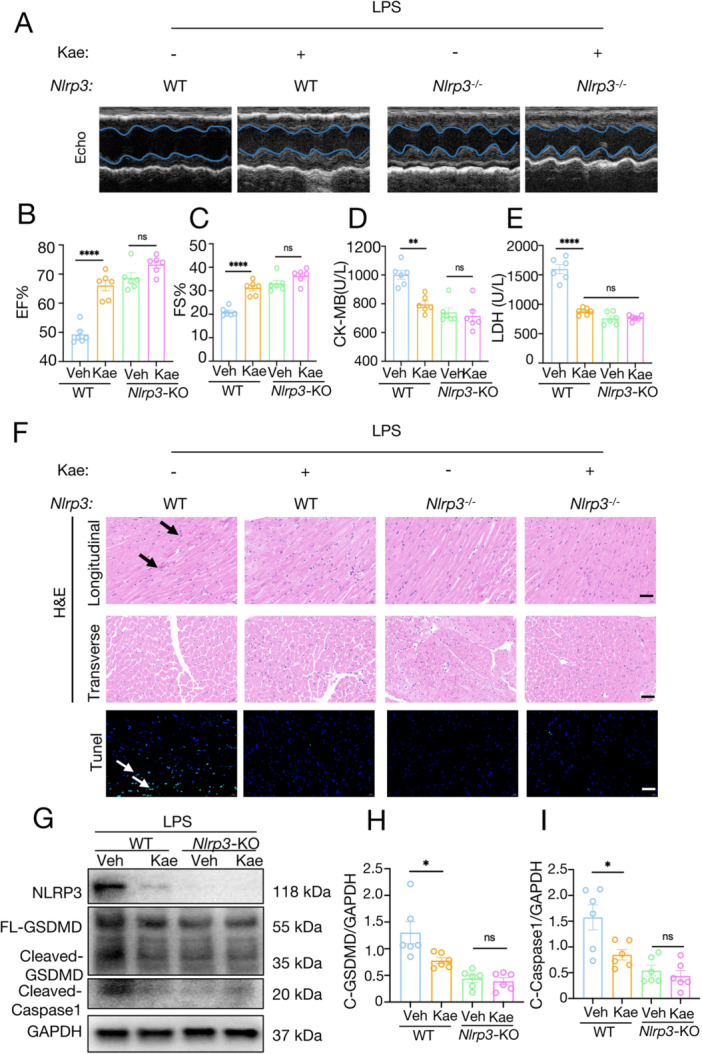
NLRP3 is required for Kae to defend against cardiotoxicity induced by LPS in vivo. The WT mice and NLRP3 knockout mice were exposed to LPS (10 mg/kg) for 12 h. (A) Representative transthoracic echocardiography M‐model images from each group. (B) EF% and (C) FS% of the mice in each group. (D, E) Serum levels of CK‐MB and LDH in mice from each group. (F) Representative images of HE staining and TUNEL staining for heart tissue. Scale bar = 50 μm. *N* = 6. (G) The relative protein levels of Cleaved‐GSDMD, Cleaved‐caspase1, and GAPDH in the heart tissue were detected using western blot assay. Densitometric quantification of C‐GSDMD (H) and C‐Caspase 1 (I) were shown. *N* = 6. (One‐way analysis of variance [ANOVA] followed by Bonferroni post hoc test for comparing multiple groups. *p < 0.05, **p < 0.01, **** *p* < 0.0001, ns = not significant).

To further clarify the in vivo mechanism by which Kae exerts its power to treat septic cardiomyopathy, we constructed NLRP3 knockout mice (*Nlrp3* ^−/−^) and applied Kae to LPS‐induced NLRP3^−/−^ mice and WT mice, respectively (Supporting Information: Figure S4). Echocardiography results showed that, consistent with previous results, Kae significantly alleviated LPS‐induced cardiac dysfunction, which could be seen in EF and FS. However, LPS‐induced cardiac dysfunction was not significant in NLRP3^−/−^ mice and did not differ from the Kae treatment group (Figure 5A–C).

In addition, we observed that Kae significantly reduced serum levels of CK‐MB and LDH in LPS‐induced mice, but we did not observe these beneficial effects with NLRP3 deletion (Figure 5D,E). Subsequently, HE staining was performed on myocardial tissues of each group, and the results indicated that Kae no longer appeared to have significant effects on inhibiting myocardial fiber rupture, myocardial cell edema, and inflammatory cell infiltration in NLRP3^−/−^ mice (Figure 5F, Supporting Information: Figure S5). Similar results were obtained in Tunnel results in myocardial tissue, where LPS‐induced apoptosis of cardiomyocytes was no longer significant after NLRP3 deletion, and there was no difference in the effect of Kae treatment on cardiomyocytes between the two groups in NLRP3^−/−^ mice. Finally, Western blot was used to detect the proteins in the myocardial tissue of mice in each group. Similar to the previous results, in the heart tissue of NLRP3^−/−^ mice, the inhibitory effect of Kae on cleaved‐GSDMD and cleaved Caspase‐1was not observed (Figure 5G,I). These results suggest that NLRP3 is necessary for Kae in the treatment of LPS‐induced septic cardiomyopathy.

## 4 Discussion

Sepsis‐induced myocardial injury is a prevalent complication that is characterized by a high risk of morbidity and mortality. Meanwhile, inflammatory cytokines such as IL‐1β, IL‐6, and TNF‐α are well‐known to be significant contributors to the pathogenesis of sepsis‐induced cardiomyopathy [7, 16, 17, 18]. The present study demonstrated that LPS resulted in a significant decline in EF and FS. The histological analyses revealed myocardial damage and apoptosis. The findings of this study were corroborated by elevated levels of inflammatory cytokines, including IL‐1β, IL‐6, IL‐18, and TNF‐α. However, Kae treatment, a potent flavonoid compound extracted from Cinnamomum osmophloeum [13], significantly reduced the levels of these inflammatory cytokines in the cardiac tissue, mitigating myocardial damage and apoptosis, and improving cardiac function in LPS‐induced mice.

Subsequent analysis of the transcriptomics data indicated the involvement of the NF‐κB signaling pathway. As has been previously confirmed by a number of studies, the NLRP3 pathway is one of the downstream pathways of NF‐κB [19, 20, 21]. The expression of Nlrp3 in the heart tissue was therefore detected, and this presented as being down‐regulated in mice treated with LPS and Kae. In addition, the study demonstrated that Kae treatment led to a reduction in the protein expression levels of p‐p65 and NLRP3 in the hearts of mice stimulated by LPS. Consequently, Kae is hypothesised to attenuate inflammation and apoptosis in myocardial cells of septic mice by inhibiting the NF‐κB/NLRP3 signaling pathway, thereby restoring damaged cardiac function.

It has been hypothesised that the NLRP3 inflammasome plays a pivotal role in cardiomyocytes [22, 23, 24]. The activation of the NLRP3 inflammasome leads to the auto‐cleavage of pro‐caspase‐1, which in turn mediates the maturation and secretion of pro‐inflammatory cytokines such as IL‐1β and IL‐18 [22, 25]. Furthermore, it has been demonstrated that caspase‐1 can cleave GSDMD, yielding an N‐terminal cleavage product (NT‐GSDMD), which has been shown to induce pyroptosis by forming plasma membrane pores [26, 27, 28]. Furthermore, the pyroptosis of cardiomyocytes was evaluated by the levels of GSDMD‐NT, cleaved‐caspase1, IL‐1β, and IL‐18 in heart tissues. The present study revealed that LPS stimulation exhibited elevated levels of NT‐GSDMD, cleaved caspase 1, IL‐1β, and IL‐18, indicating an increase in cell pyroptosis both in vitro and in vivo. It was observed that Kae was capable of attenuating the LPS‐induced upregulation of these markers in mouse heart tissues. In addition, the inhibitory effect of Kae on LPS‐induced myocardial inflammation was corroborated through in vitro experimentation, utilising the NF‐κB/NLRP3/pyroptosis pathway. Also, it showed that Kae was applied to LPS‐induced NLRP3^−/−^ mice and WT mice, and determined that the loss of function of the NLRP3 gene resulted in a significant reduction in the damage to cardiomyocytes caused by LPS. Furthermore, the effect of Kae treatment on cardiomyocytes was found to be similar in NLRP3^−/−^ mice and in the control group. These findings suggest that the NLRP3 is necessary for the treatment of LPS‐induced septic cardiomyopathy using Kae.

**Figure 6:**
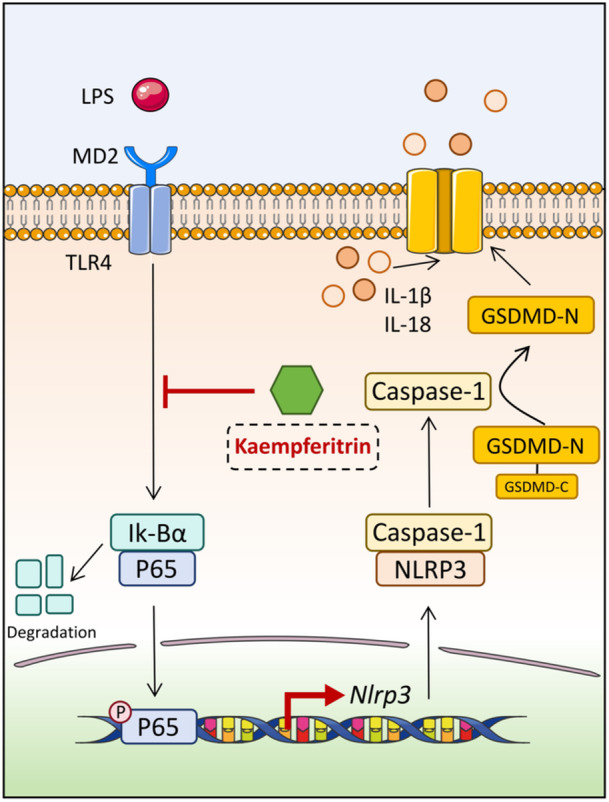
The graphic abstract shows that Kae could alleviate myocardial damage associated with sepsis via the NF‐κB/NLRP3/pyroptosis pathway.

The research further discovered that Kae could alleviate myocardial damage associated with sepsis to a certain degree via the NF‐κB/NLRP3/pyroptosis pathway, as illustrated in Figure 6. Consequently, for patients with clinical sepsis, the appropriate use of Bupleurum as an adjunctive therapy may effectively mitigate myocardial inflammatory damage and improve cardiac function. However, further clinical data is required to substantiate these findings.

## Author Contributions

**Hongyu Kuang:** conceptualization, data curation, formal analysis, investigation, software, supervision, visualization, writing – original draft, revised manuscript, writing‐reviewing and editing. **Qiang Li:** conceptualization, data curation, formal analysis, investigation, methodology, validation, writing – original draft, revised manuscript. **Min Chen:** conceptualization, data curation, investigation, methodology, validation, visualization. **Huaan Du:** conceptualization, methodology, supervision, writing – reviewing and editing.

## Ethics Statement

The animal experimental procedures were approved by the Ethics Committee of the Animal Experimentation Center of Chongqing Medical University.

## Conflicts of Interest

The authors declare no conflicts of interest.

## Supporting information


**Figure S1:** A‐B Levels of IL‐6 (A) and TNF‐α (B) in heart tissue detected by ELISA assay. **Figure S2:** The qualified tunnel assay for Figure 1G. **Figure S3:** Bright field of H9c2 incubated with Kae at different concentration from 0 to 50μm. **Figure S4:** To investigate the role of NLRP3, we constructed Nlrp3‐/‐ mice, which were genotyped by PCR. **Figure S5:** The qualified tunnel assay for Figure 5F. **Table S1:** Primer sequences used for real‐time qPCR analysis. **Table S2:** Echocardiographic parameters.

Supplementary of Original blots.

Supplementary_of_Original_blots

## Data Availability

Data and materials are available if needed by contacting the corresponding author.
