# Supplementary material for: Kaempferitrin Attenuates Lipopolysaccharide‐Induced Cardiac Dysfunction Through Suppression of the NF‐κB/NLRP3 Signaling Pathway
Source: Immun Inflamm Dis. 2026 Jan 26;14(1):e70323. doi: 10.1002/iid3.70323 (PMC12835615; doi:10.1002/iid3.70323)
Supplement: Supplementary file 1 — Figure S1: A‐B Levels of IL‐6 (A) and TNF‐α (B) in heart tissue detected by ELISA assay. Figure S2: The qualified tunnel assay for Figure 1G. Figure S3: Bright field of H9c2 incubated with Kae at different concentration from 0 to 50μm. Figure S4: To investigate the role of NLRP3, we constructed Nlrp3‐/‐ mice, which were genotyped by PCR. Figure S5: The qualified tunnel assay for Figure 5F. Table S1: Primer sequences used for real‐time qPCR analysis. Table S2: Echocardiographic parameters. [file IID3-14-e70323-s002.pdf]

Kaempferitrin Attenuates Lipopolysaccharide-Induced Cardiac Dysfunction Through Suppression of the NF-κB/NLRP3 Signaling Pathway

Supplemental Material

Contents in Supplementary File

Supplementary Material and Methods 2 Tables, 5 Figures and Legends

Table S1 Primer sequences used for real-time qPCR analysis

Gene	Species	Sequence (Forward)	Sequence (Reverse)
IL-1β	Rat	AAGCTCTCCACCTCAATGGAC	GTGCCGTCTTTCATCACACAG
IL-6	Rat	AGCCAGAGTCATTCAGAGCA	TGGTCTTGGTCCTTAGCCAC
TNF-α	Rat	GGCGTGTTCATCCGTTCTC	CTTCAGCGTCTCGTGTGTTTCT
GAPDH	Rat	GGCACAGTCAAGGCTGAGAATG	ATGGTGGTGAAGACGCCAGTA
IL-1β	Human	CTGTCCTGCGTGTTGAAAGATG	TCTGCTTTTGAGAGGTGCTGATG
IL-6	Human	TGGCAGAAAACAACCTGAACC	ACCTCCAAACTCCAAAAGACCAG
TNF-α	Human	CTGCCTGCTGCACTTTGGAG	ACATGGGCTACAGGCTTGTCACT
GAPDH	Human	GCACCGTCAAGGCTGAGAAC	TGGTGAAGACGCCAGTGGA

Table S2 Echocardiographic parameters

Parameters	Control (n=6)	Kae (n=6)	LPS (n=6)	LPS+Kae (10mg/kg, n=6)	LPS+Kae (20mg/kg, n=6)
LVPWT;s, (mm)	1.29±0.12	1.18±0.15	0.68±0.042	1.03±0.13	1.11±0.09
LVPWT;d, (mm)	0.85±0.081	0.78±0.049	0.60±0.19	0.73±0.10	0.75±0.056
IVST;s, (mm)	1.21±0.15	1.18±0.11	0.88±0.22	1.12±0.13	1.16±0.078
IVST;d, (mm)	0.81±0.076	0.75±0.082	0.50±1.34	0.75±0.031	0.76±0.055
LVIDs, (mm)	2.01±0.098	1.93±0.21	2.35±0.10	2.28±0.23	2.09±0.36
LVIDd, (mm)	3.29±0.15	3.25±0.30	3.08±0.20	3.24±0.30	3.26±0.53
LVESV (ml)	0.022±0.004	0.020±0.006	0.035±0.005	0.033±0.01	0.025±0.01
LVEDV (ml)	0.09±0.011	0.09±0.024	0.073±0.014	0.088±0.023	0.092±0.041

Kae=Kaempferitrin, LPS=Lipopolysaccharide, LVPWT= Left ventricular posterior wall thickness, IVST= Interventricular septal wall thickness, LVID=Left ventricular internal diameter, LVESV=Left ventricular end-systolic volume, LVEDV=Left ventricular end-diastolic volume

Supplementary Figure 1

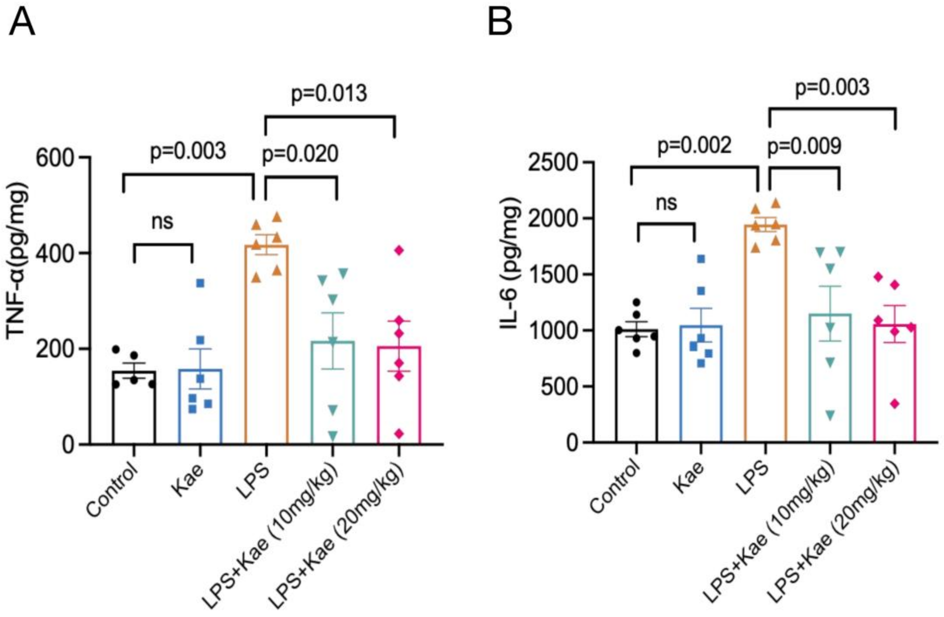

Figure S1A-B Levels of IL-6 (A) and TNF-α (B) in heart tissue detected by ELISA assay.

<u>Supplementary Figure 2</u>

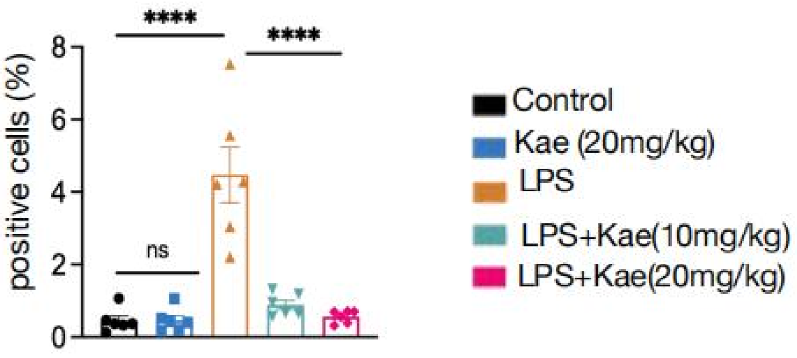

Figure S2 The qualified tunnel assay for Figure 1G.

Supplementary Figure 3

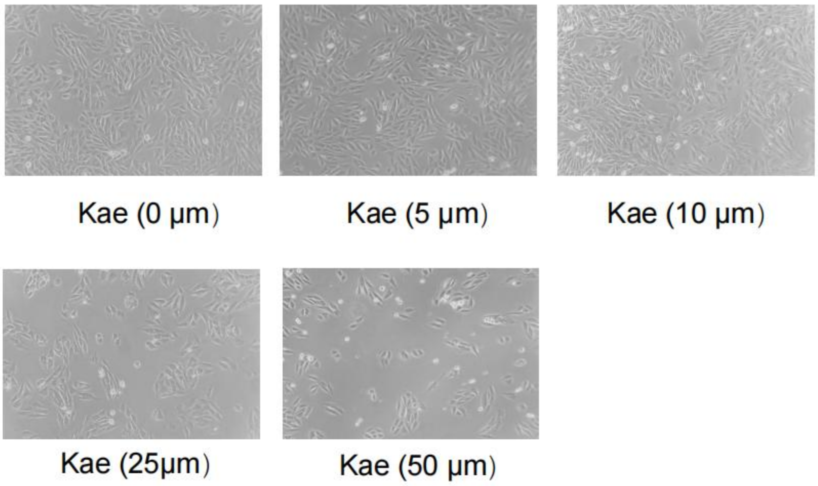

Figure S3 Bright field of H9c2 incubated with Kae at different concentration from 0 to 50μm。

Supplementary Figure 4

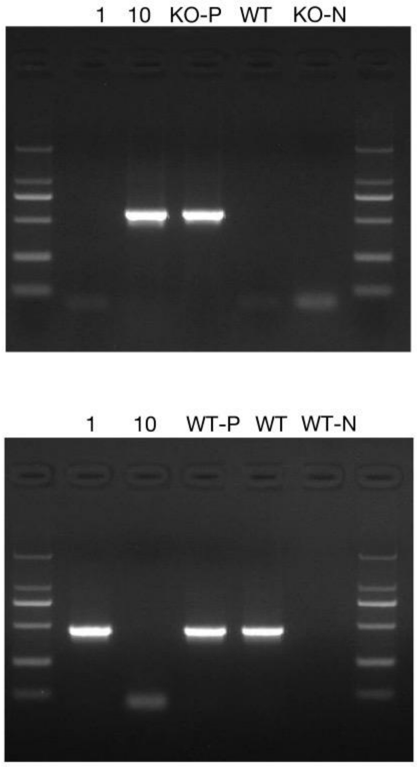

Fig S4 To investigate the role of NLRP3, we constructed *Nlrp3*$^{-/-}$ mice, which were genotyped by PCR.

Supplementary Figure 5

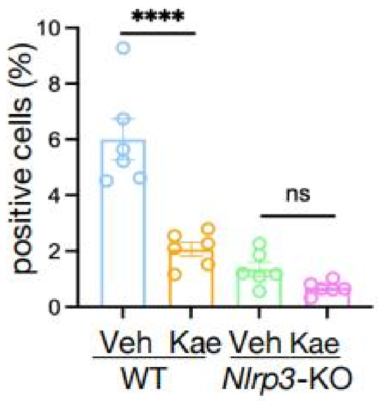

Figure S5. The qualified tunnel assay for Figure 5F.
