# Supplementary material for: Kaempferitrin Attenuates Lipopolysaccharide‐Induced Cardiac Dysfunction Through Suppression of the NF‐κB/NLRP3 Signaling Pathway
Source: Immun Inflamm Dis. 2026 Jan 26;14(1):e70323. doi: 10.1002/iid3.70323 (PMC12835615; doi:10.1002/iid3.70323)

Original blots for Figure 2G

Repeat1

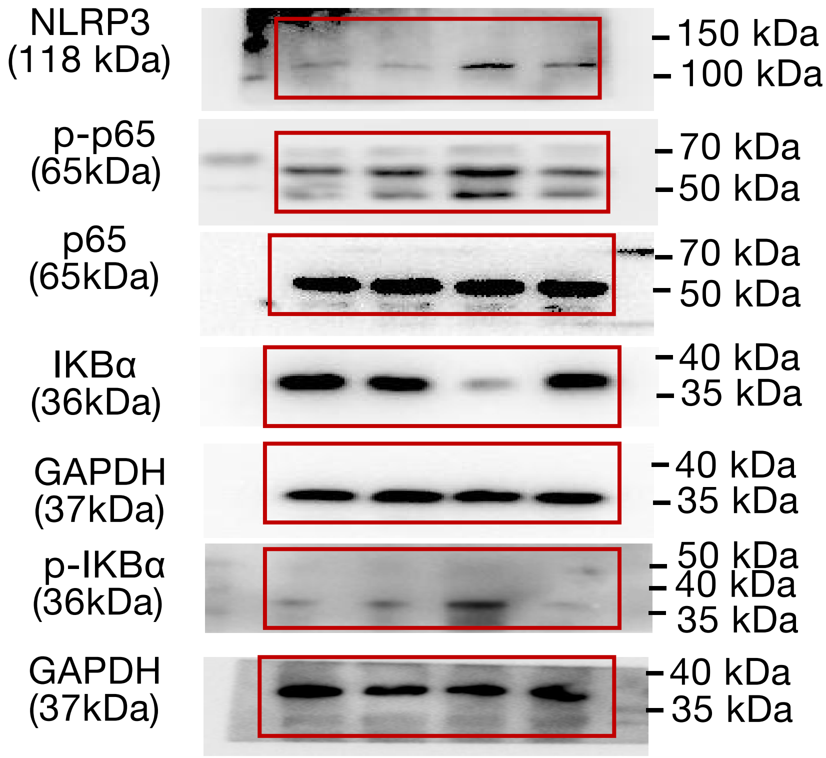

Original blots for Figure 2G

Repeat 2

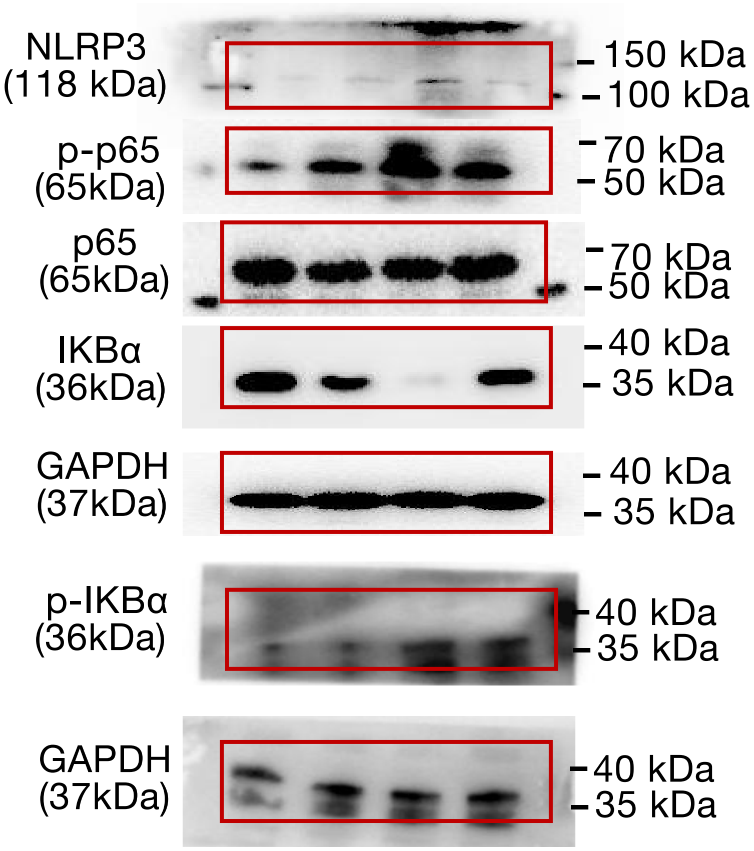

Original blots for Figure 2G

Repeat 3

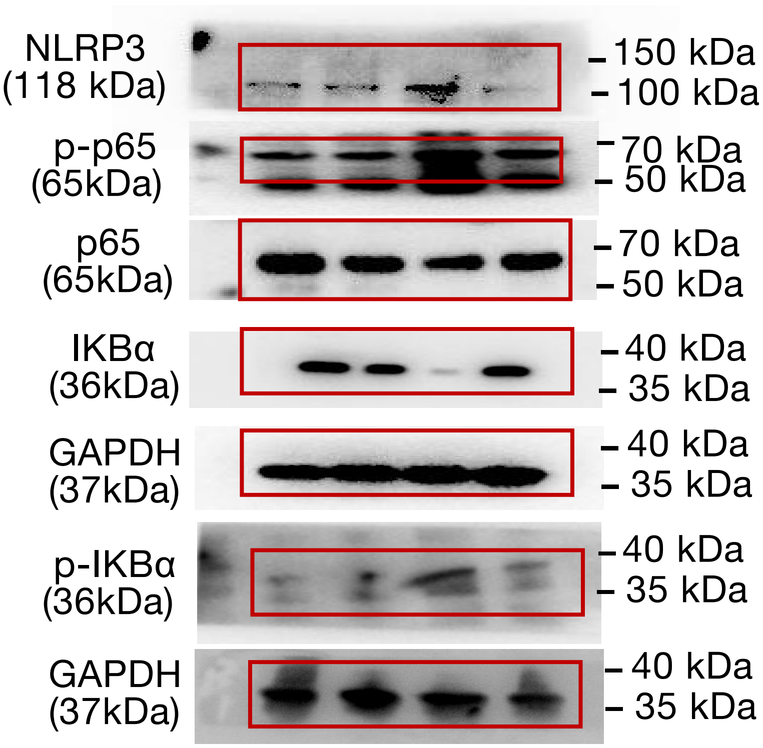

Original blots for Figure 2J

Repeat1

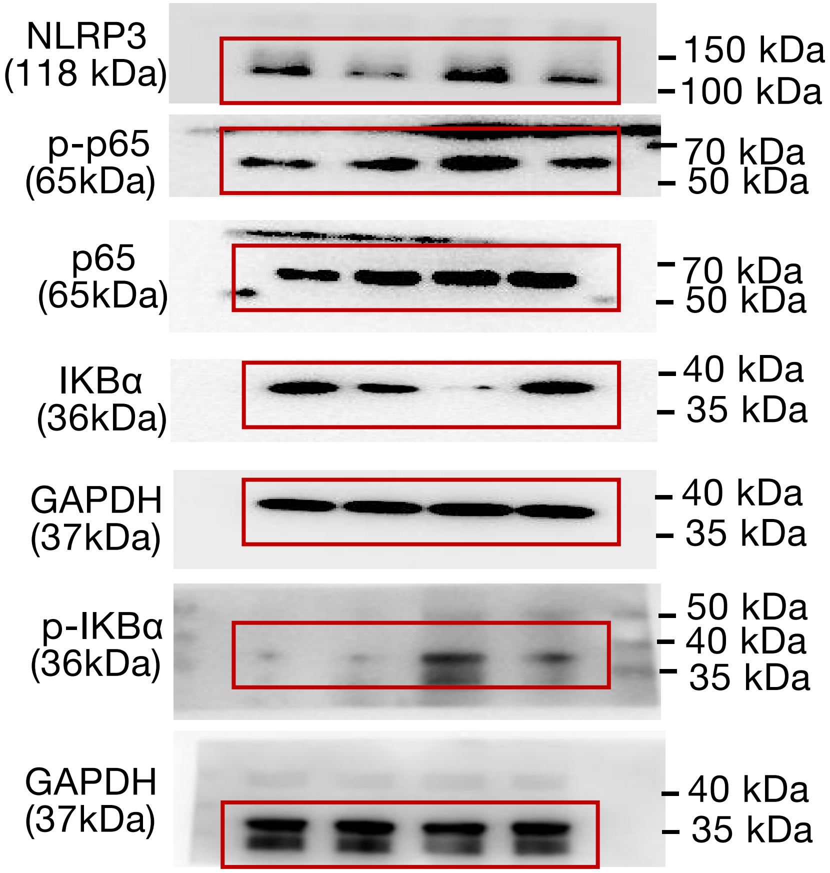

Original blots for Figure 2J

Repeat 2

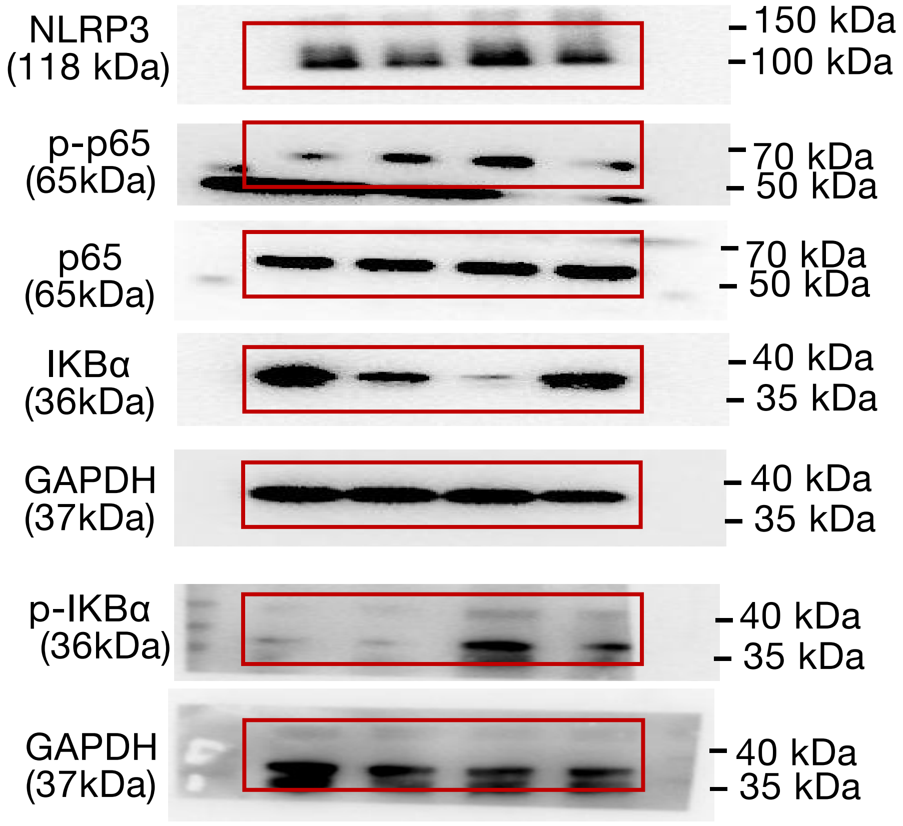

Original blots for Figure 2J

Repeat 3

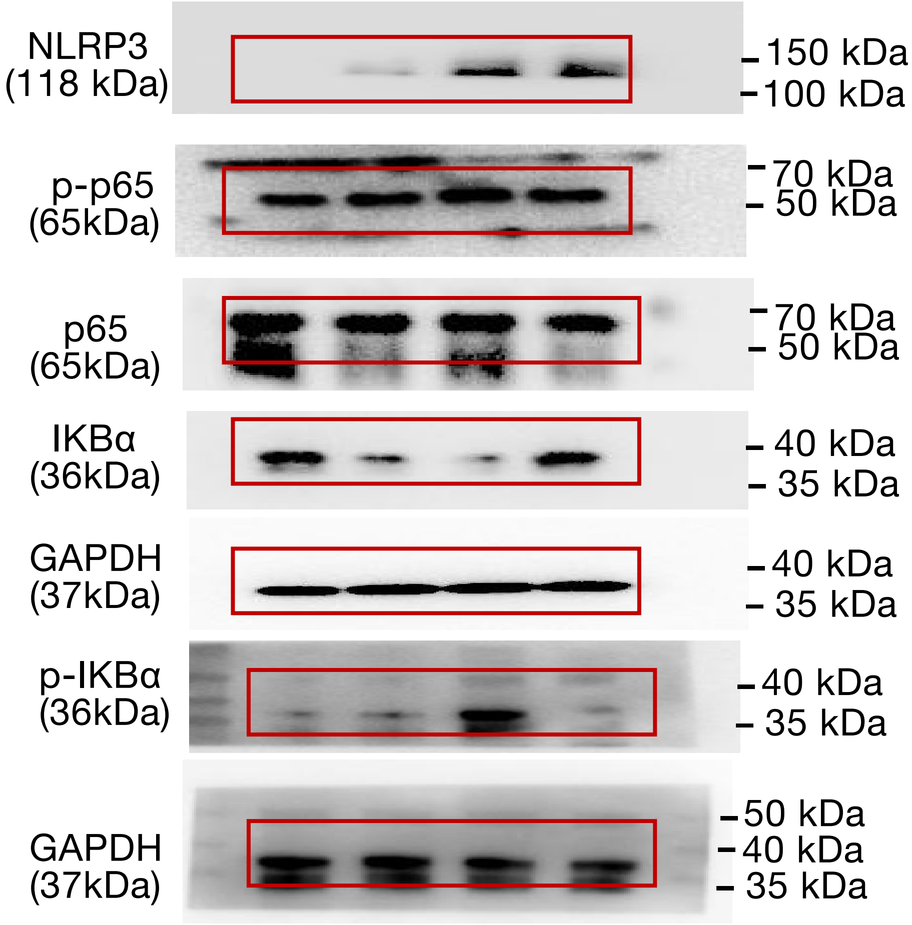

Original blots for Figure 2L

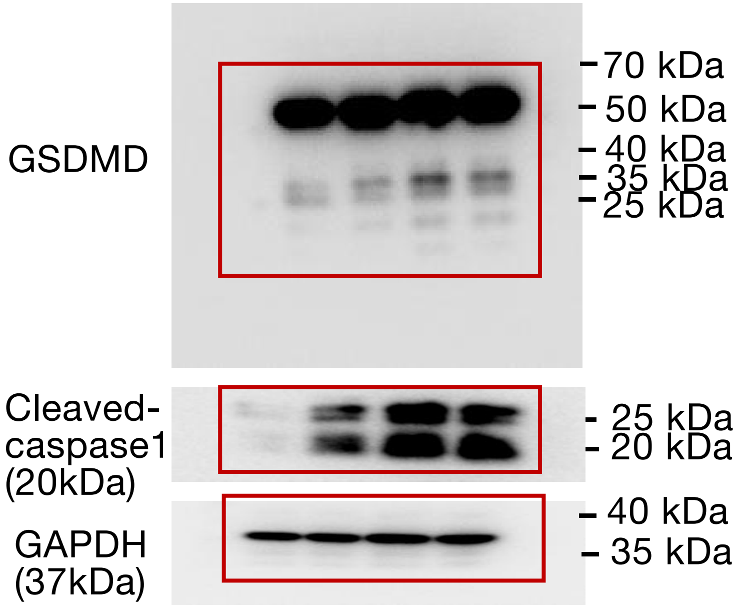

Original blots for Figure 2J

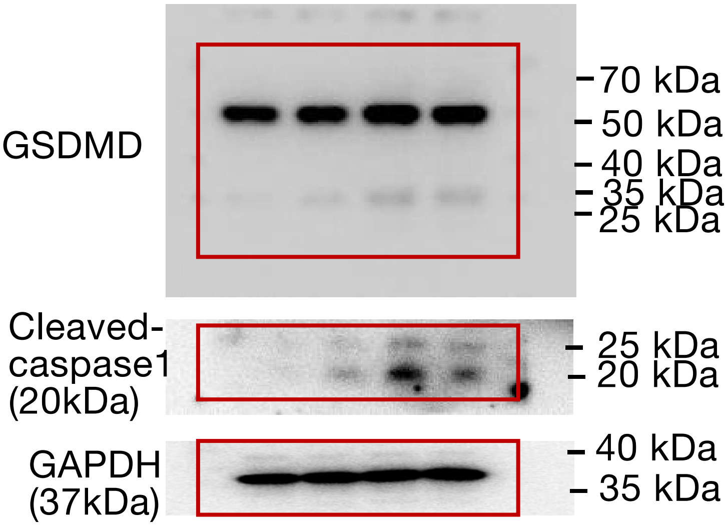

Original blots for Figure 3A

Repeat 1

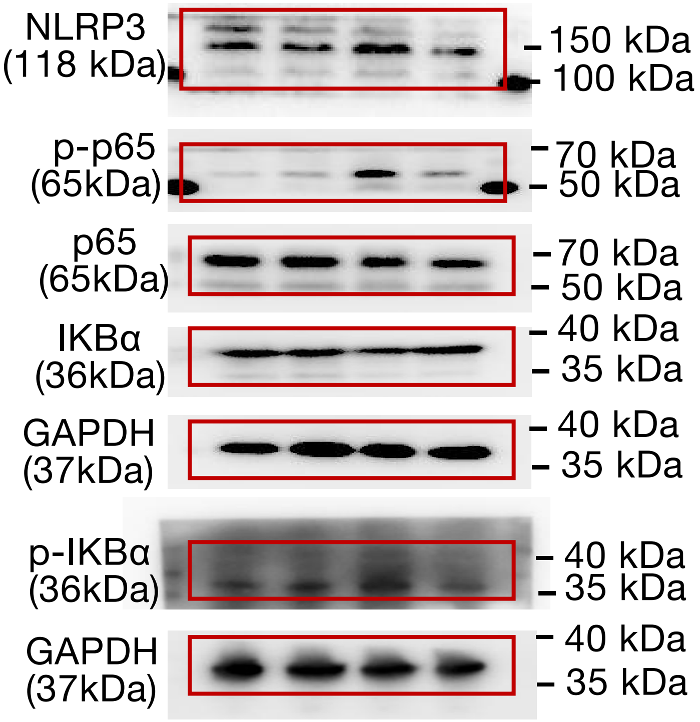

Original blots for Figure 3A

Repeat 2

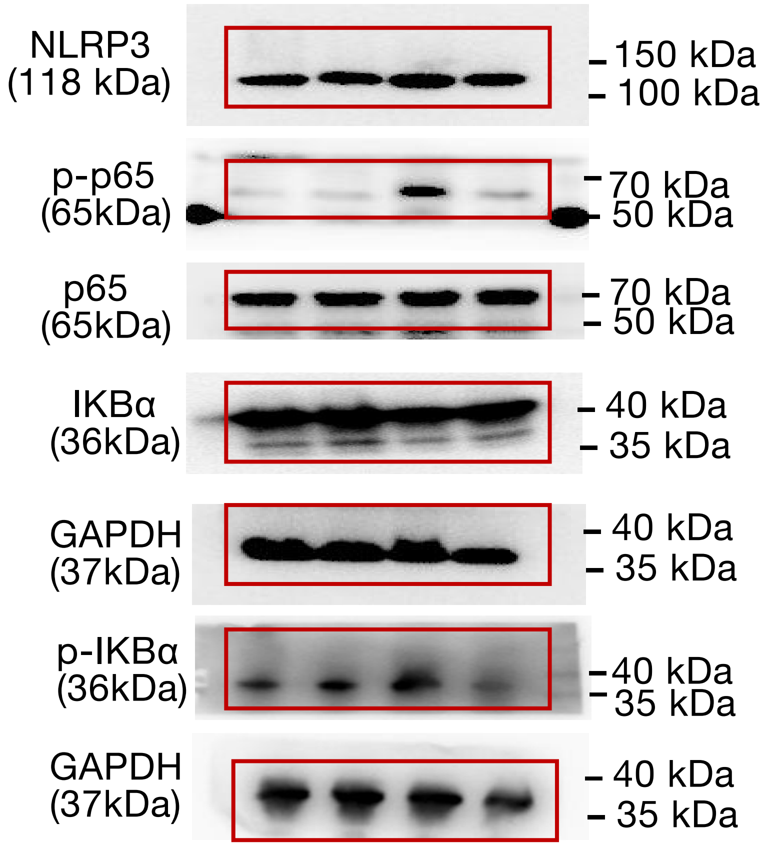

Original blots for Figure 3A

Repeat 3

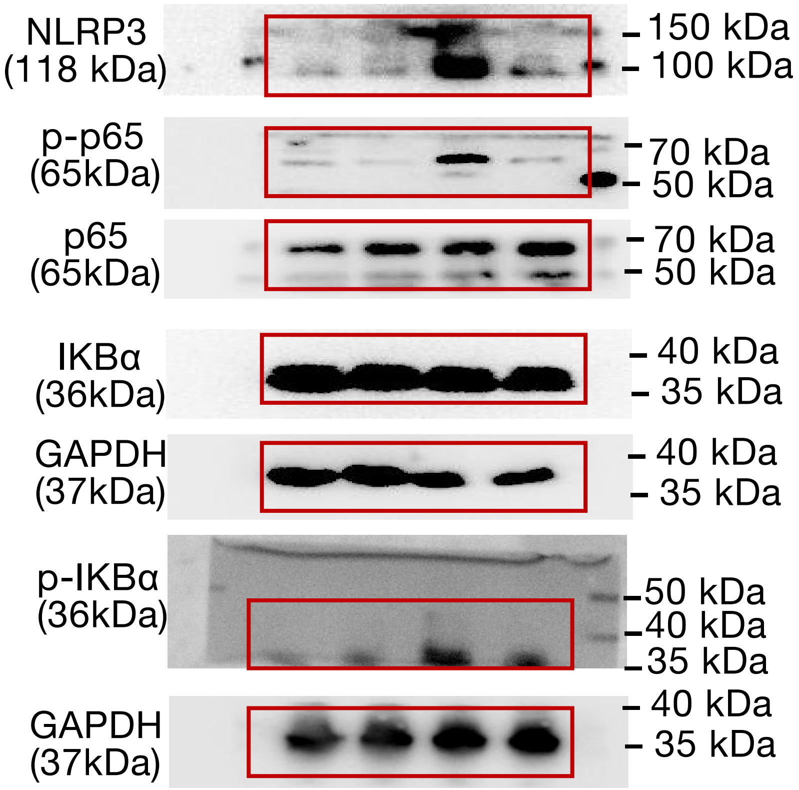

Original blots for Figure 3B

Repeat 1

GSDMD
(Long exposure)

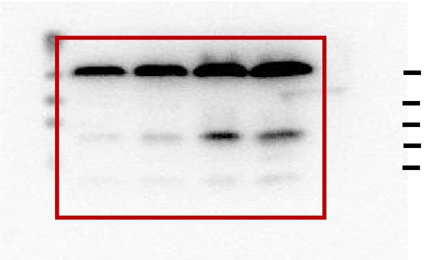

70 kDa
50 kDa
40 kDa
35 kDa
25 kDa

GSDMD
(Short exposure)

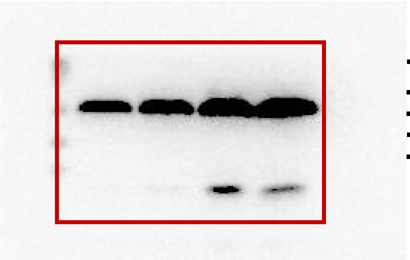

70 kDa
50 kDa
40 kDa
35 kDa
25 kDa

GAPDH
(37kDa)

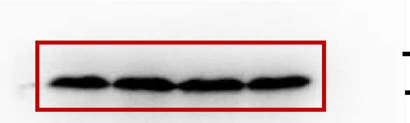

40 kDa
35 kDa

Cleaved-
caspase1
(20kDa)

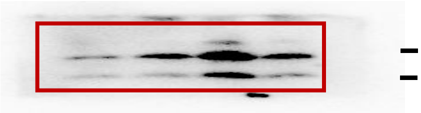

25 kDa
20 kDa

Original blots for Figure 3B

Repeat 2

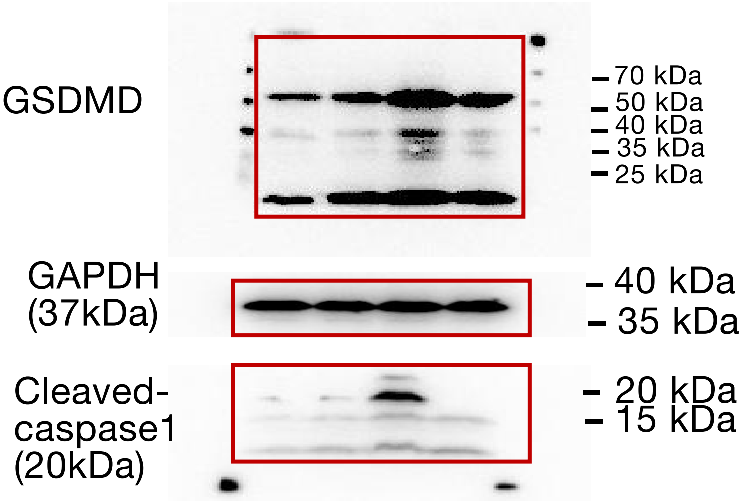

Original blots for Figure 3B

Repeat 3

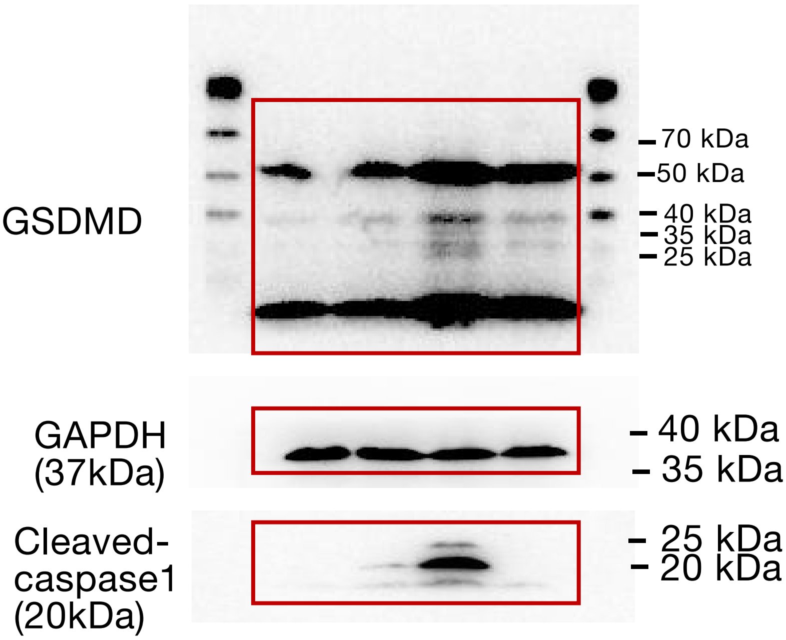

Original blots for Figure 4A

Repeat 1

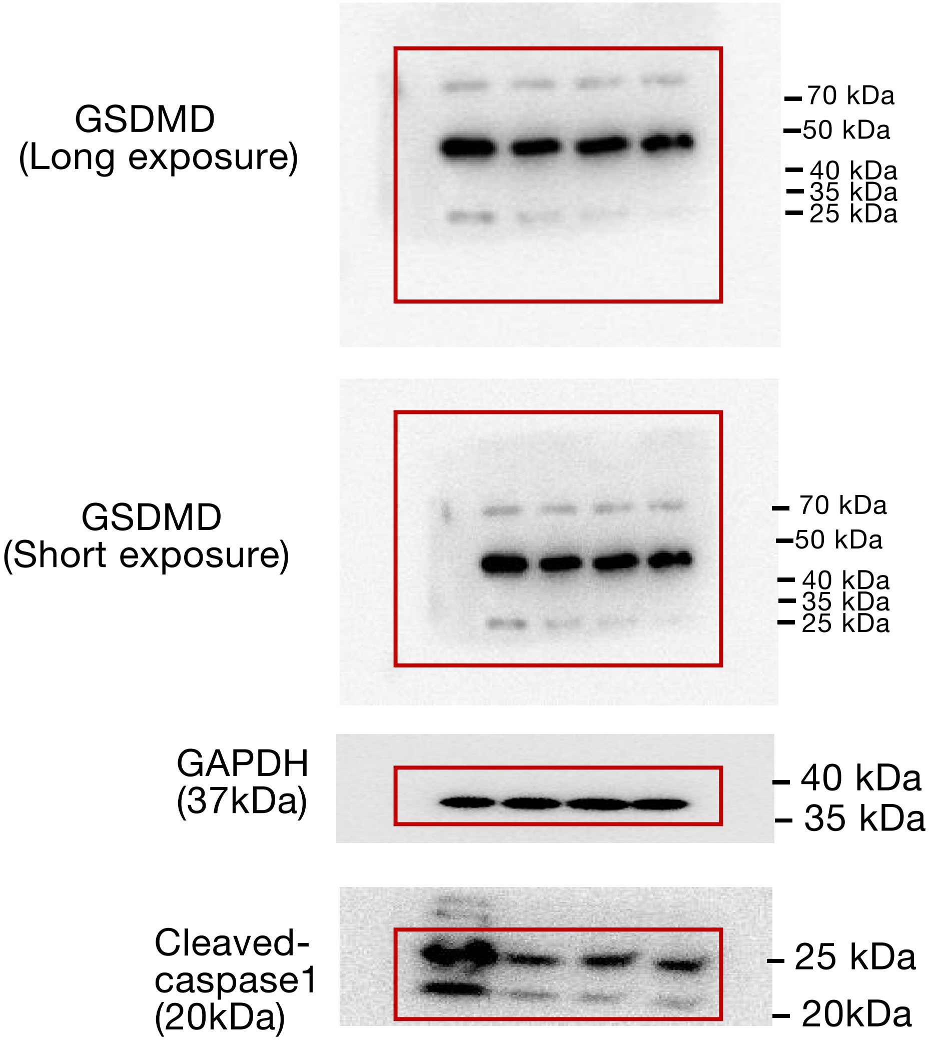

Original blots for Figure 4A

Repeat 2

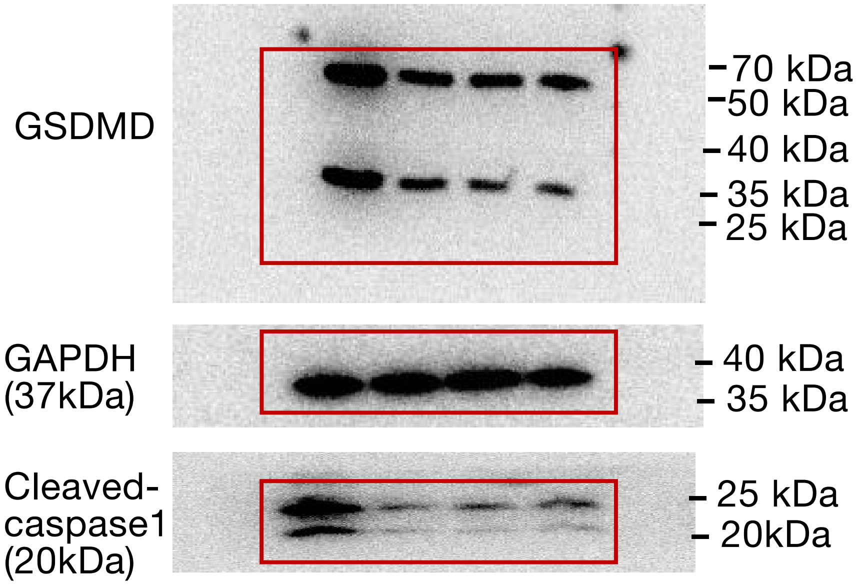

Original blots for Figure 4A

Repeat 3

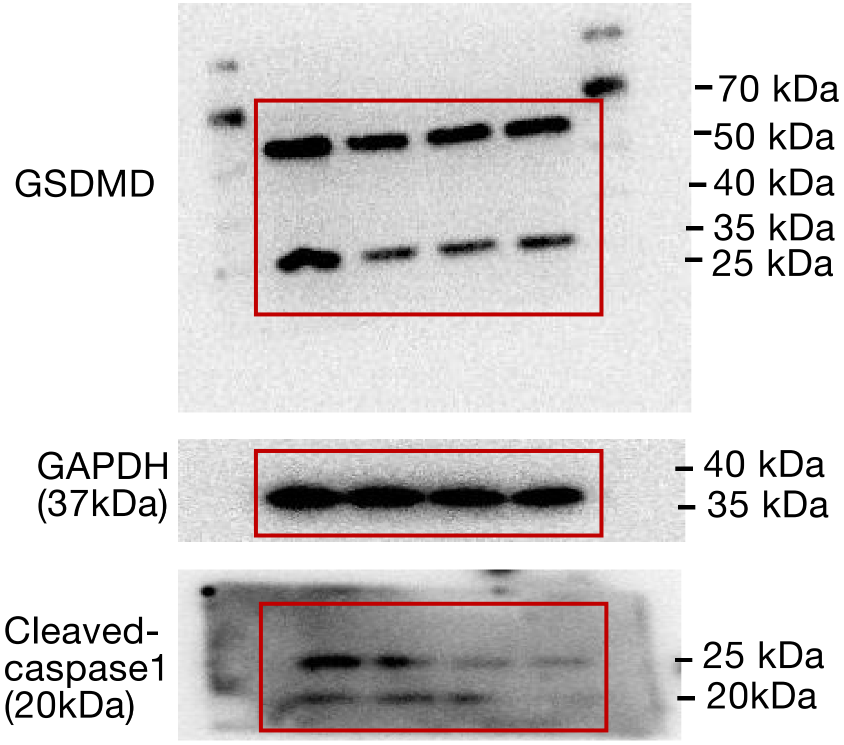

Original blots for Figure 4E

Repeat 1

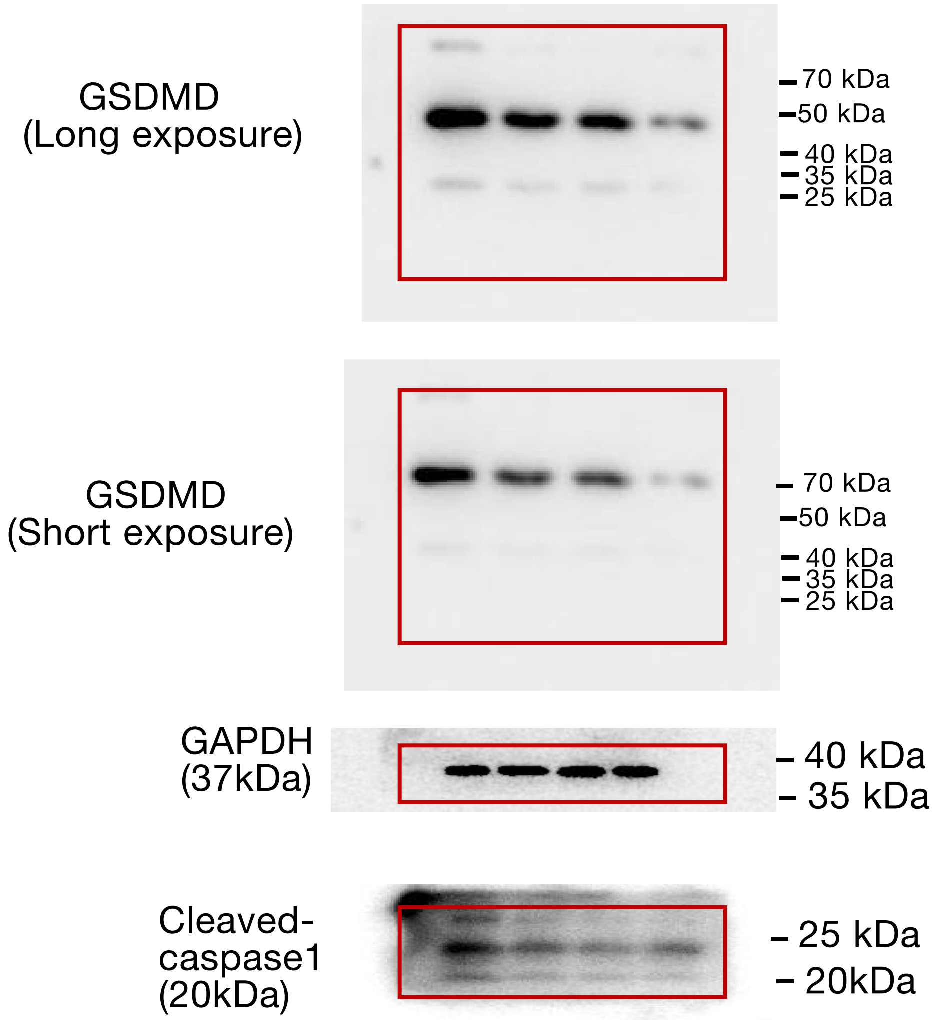

Original blots for Figure 4E

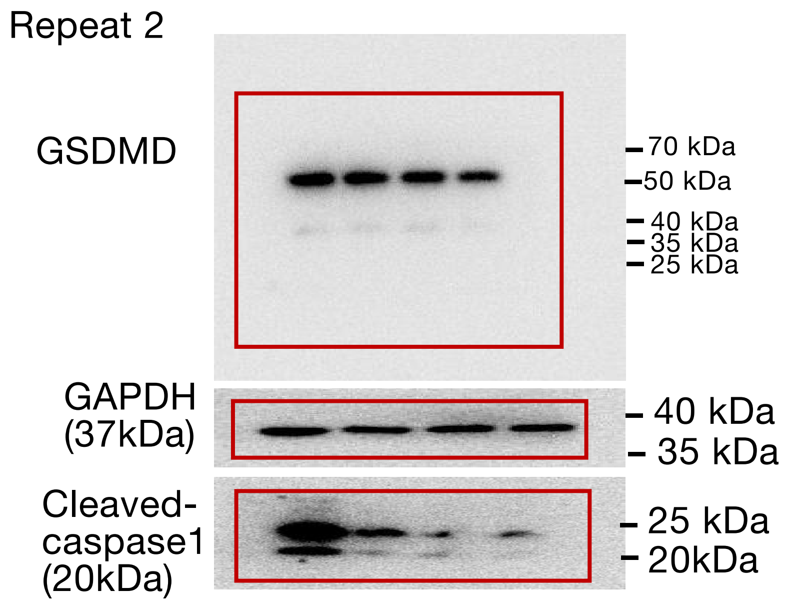

Original blots for Figure 4E

Repeat 3

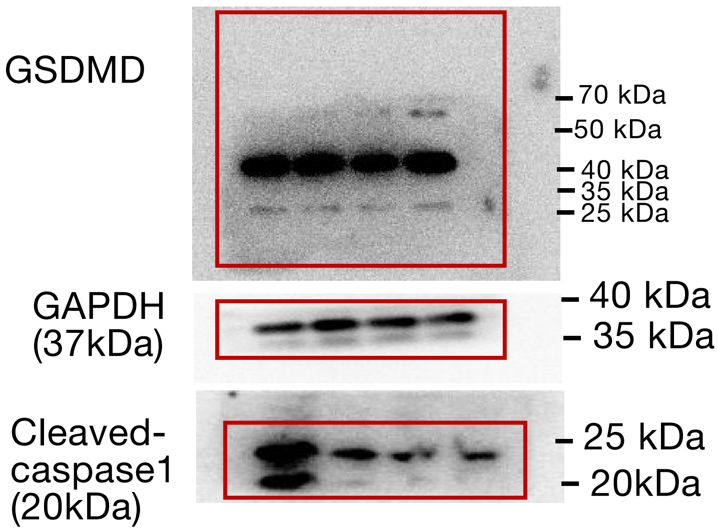

Original blots for Figure 5G

Repeat 1

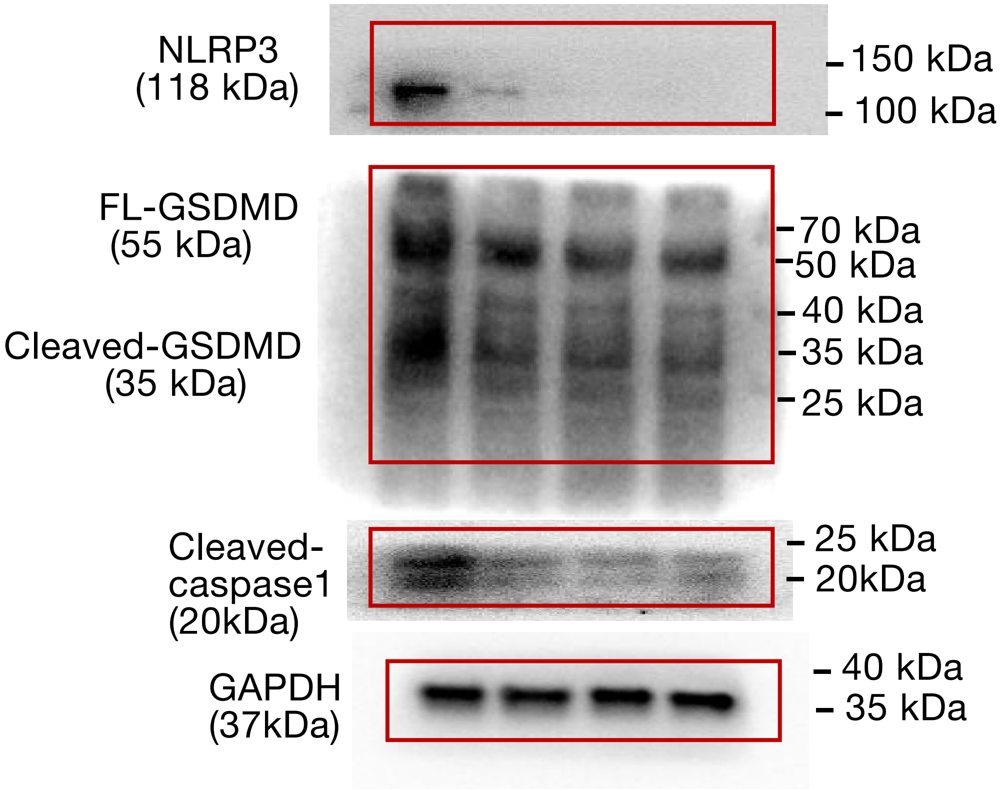

Original blots for Figure 5G

Repeat 2

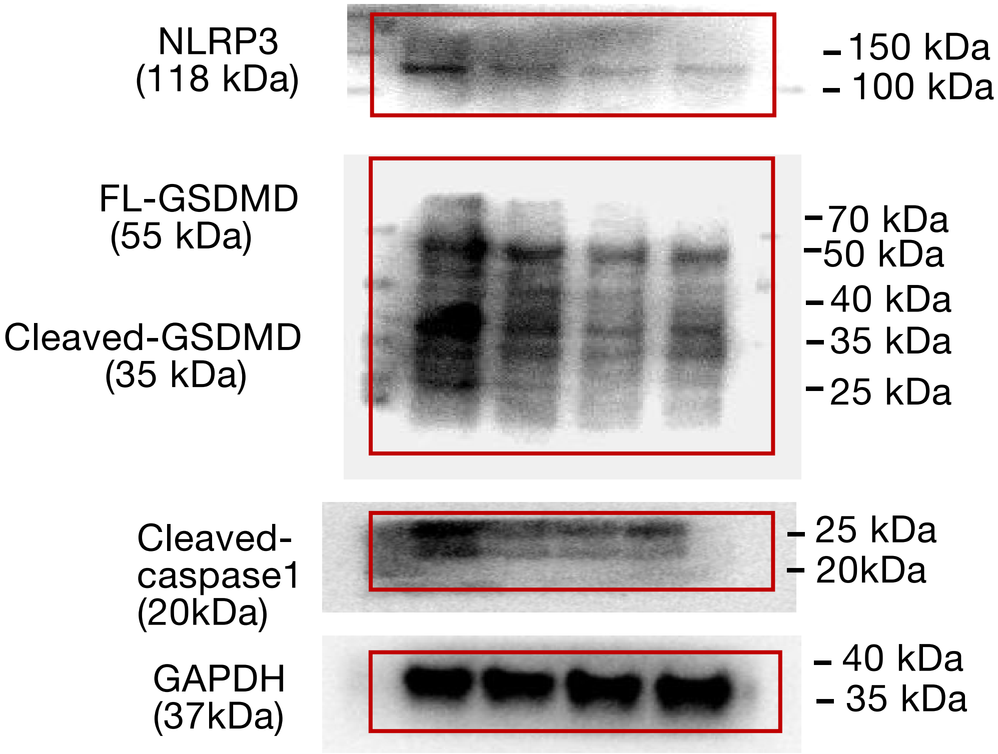

Original blots for Figure 5G

Repeat 3

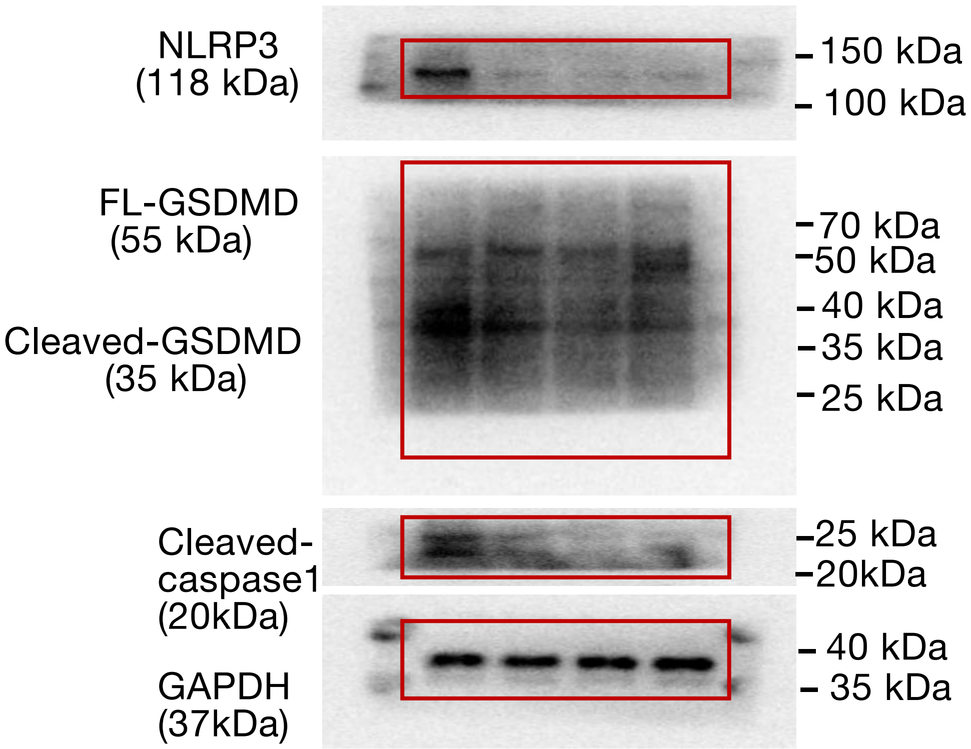

Supplement: Supplementary file 2 — Supplementary of Original blots. [file IID3-14-e70323-s003.pdf]
