# Supplementary material for: Kaempferitrin Attenuates Lipopolysaccharide‐Induced Cardiac Dysfunction Through Suppression of the NF‐κB/NLRP3 Signaling Pathway
Source: Immun Inflamm Dis. 2026 Jan 26;14(1):e70323. doi: 10.1002/iid3.70323 (PMC12835615; doi:10.1002/iid3.70323)
Supplement: Supplementary file 3 — Supplementary_of_Original_blots [file IID3-14-e70323-s001.docx]

**Supplementary**

**Table S1 Echocardiographic parameters**

| **Parameters** | **Control   (n=6)** | **Kae  (n=6)** | **LPS  (n=6)** | **LPS+Kae  (10mg/kg, n=6)** | **LPS+Kae (20mg/kg, n=6)** |
| --- | --- | --- | --- | --- | --- |
| LVPWT;s, (mm) | 1.29±0.12 | 1.18±0.15 | 0.68±0.042 | 1.03±0.13 | 1.11±0.09 |
| LVPWT;d, (mm) | 0.85±0.081 | 0.78±0.049 | 0.60±0.19 | 0.73±0.10 | 0.75±0.056 |
| IVST;s, (mm) | 1.21±0.15 | 1.18±0.11 | 0.88±0.22 | 1.12±0.13 | 1.16±0.078 |
| IVST;d, (mm) | 0.81±0.076 | 0.75±0.082 | 0.50±1.34 | 0.75±0.031 | 0.76±0.055 |
| LVIDs, (mm) | 2.01±0.098 | 1.93±0.21 | 2.35±0.10 | 2.28±0.23 | 2.09±0.36 |
| LVIDd, (mm) | 3.29±0.15 | 3.25±0.30 | 3.08±0.20 | 3.24±0.30 | 3.26±0.53 |
| LVESV (ml) | 0.022±0.004 | 0.020±0.006 | 0.035±0.005 | 0.033±0.01 | 0.025±0.01 |
| LVEDV (ml) | 0.09±0.011 | 0.09±0.024 | 0.073±0.014 | 0.088±0.023 | 0.092±0.041 |

Kae=Kaempferitrin, LPS=Lipopolysaccharide, LVPWT= Left ventricular posterior wall thickness, IVST= Interventricular septal wall thickness, LVID=Left ventricular internal diameter, LVESV=Left ventricular end-systolic volume, LVEDV=Left ventricular end-diastolic volume
